# Aspirin Disrupts the Crosstalk of Angiogenic and Inflammatory Cytokines between 4T1 Breast Cancer Cells and Macrophages

**DOI:** 10.1155/2018/6380643

**Published:** 2018-06-24

**Authors:** Chia-Chien Hsieh, Chih-Hsuan Wang

**Affiliations:** Department of Human Development and Family Studies, National Taiwan Normal University, Taipei, Taiwan

## Abstract

The tumor microenvironment is rich in multiple cell types that influence tumor development. Macrophages infiltrate tumors, where they are the most abundant immune cell population and secrete a number of cytokines. Aspirin acts as a chemopreventive agent against cancer development. This study investigated whether aspirin regulates crosstalk between breast cancer cells and macrophages. To study these interactions in a tumor microenvironment, a conditioned media was employed using 4T1 breast cancer cells cultured in RAW 264.7 cell-conditioned medium (RAW-CM), and a cocultured model of both cells was used. When 4T1 cells were cultured in the RAW-CM, there were increases in cell viability and secretion of the cytokines VEGF, PAI-1, TNF-*α*, and IL-6. Treatment with aspirin inhibited 4T1 cell growth and migration and MCP-1, PAI-1, and IL-6 production. In the coculture of both cells, aspirin inhibited secretion of MCP-1, IL-6, and TGF-*β*. Furthermore, aspirin significantly decreased the M2 macrophage marker CD206, but increased M1 marker CD11c expression. In summary, aspirin treatment inhibited the crosstalk of 4T1 and RAW 264.7 cells through regulation of angiogenic and inflammatory mediator production and influenced the M1/M2 macrophage subtype. This highlighted that aspirin suppresses the tumor favorable microenvironment and could be a promising agent against triple-negative breast cancer.

## 1. Introduction

Breast cancer is the most frequently occurring cancer in women worldwide, especially in developed countries, and the incidence is increasing globally. In 2015, the World Health Organization performed a statistical analysis that revealed approximately 570,000 women die from breast cancer annually, indicating that up to 15% of all deaths in women are due to cancer [1]. Breast cancer has a heterogeneous pathology comprised of multiple components, including tumor cells and neighboring stromal cells, such as adipocytes, fibroblasts, macrophages, and other immune cells, that play fundamental roles in normal mammary development as well as breast carcinogenesis [2, 3]. Moreover, tumor microenvironment changes, such as changes in the extracellular matrix, soluble factors, and signaling molecules, stimulate carcinogenesis and resistance to the immune response [2]. These diverse microenvironments play critical roles in tumor progression and metastasis.

The complicated interactions between tumors and the immune system have attracted the attention of scientists over the past decade. Briefly, the dynamic interactions between innate and adaptive immunity play an important role in tumor progression and inhibition [4]. Mononuclear phagocytes are innate immune cells that protect individuals from harmful pathogens and repair injured tissues. However, in the tumor microenvironment, malignancies recruit circulating monocytes by producing tumor-derived chemotactic factors such as macrophage chemoattractant protein-1 (MCP-1), vascular endothelial growth factor (VEGF), and macrophage colony-stimulating factor (MCSF) and then induce monocytes to differentiate into tumor-associated macrophages (TAMs) [5]. In the tumor microenvironment, multiple mediators are secreted and contribute to cell proliferation, migration, angiogenesis, remodeling of endothelial cells [4], providing favorable conditions for tumor growth and metastasis, and suppression of adaptive immunity [6].

Macrophages that produce mediators are crucial initiators of chronic inflammation in the tumor microenvironment. Macrophage heterogeneity includes categorization into M1 and M2 macrophages based on two distinct phenotypes that are a result of macrophage polarization and the development of different characteristics [7]. M1 macrophages produce inflammatory cytokines that evoke the adaptive immune response. Conversely, M2 macrophages promote angiogenesis and wound healing and suppress the adaptive immune responses [7]. Interestingly, TAMs resemble M2 macrophages and have protumor properties in tumor microenvironments. Several studies on *murine* tumor models have shown that TAMs promote tumors [8] and produce cytokines and chemokines that sustain and amplify the inflammatory state [9]. Therefore, agents with the potential to adjust this microenvironment have been proposed as effective future cancer therapies [3, 8].

Aspirin, acetylsalicylic acid, is a nonsteroidal anti-inflammatory drug commonly used to reduce inflammation and prevent heart attack and stroke [10, 11]. However, over the past two decades, studies have shown that regular use of aspirin may have an additional promising role against cancers [12]. This chemoprevention by aspirin was reported for inflammation-associated cancers such as colorectal, breast, lung, prostate, stomach, and ovarian cancers [10]. Moreover, accumulating epidemiological evidence has revealed that aspirin has effects when used against breast cancer [13, 14]. Although aspirin is a promising chemopreventive agent, gastrointestinal side effects and optimal doses are important factors to consider for clinical applications. Therefore, alternatives using aspirin, such as lower doses or combinations with treatments, have been continually proposed.

Currently, little is known about the role of aspirin in immune regulation of tumors, especially in terms of the tumor microenvironment. The main goal of this study was to better understand breast cancer chemoprevention by aspirin, which may regulate immune responses in both malignant cells and macrophages in the tumor microenvironment, as well as interfere with crosstalk between these cells. These insights might provide potential strategies for ameliorating triple-negative breast cancer, such as 4T1 cells, which is a highly aggressive type of breast cancer with resistance to treatments [15].

## 2. Materials and Methods

### 2.1. Cell Culture and Treatments

The murine breast cancer 4T1 cell line was purchased from the American Type Culture Collection (Manassas, VA, USA), and macrophage RAW 264.7 cell line was purchased from Bioresource Collection and Research Center (BCRC, Hsinchu, Taiwan). Both cell lines were cultured in Dulbecco's modified Eagle's medium (DMEM, Caisson, Smithfield, UT, USA) containing 10% fetal bovine serum (FBS, Genedirex, Las Vegas, NV, USA) with 1% penicillin/streptomycin/amphotericin B (Caisson) in a humidified atmosphere with 5% CO_2_ in a 37°C incubator. Both cell lines were used to prepare conditioned medium and cocultures in this study. Aspirin (Sigma, St. Louis, MO, USA) was dissolved in dimethyl sulfoxide (DMSO, Sigma) to generate a stock solution. The final concentration of DMSO in the vehicle group was 0.1%, which is equivalent to the highest dose (2 mM) received by cells during aspirin treatment.

### 2.2. RAW-CM Preparation

RAW 264.7 cells, 2.5 × 10^4^ cells/well, were seeded in 6-well plates containing 10% FBS/DMEM and cultured overnight. The cells were then cultured for 24 h in the presence or absence of 100 ng/mL lipopolysaccharide (LPS, Sigma) in 1% FBS/DMEM according to a previous study, with modifications [16]. Supernatants were collected, and cell debris was removed by centrifugation prior to use in experiments.

### 2.3. Cell Viability Assay

The 4T1 cells were seeded into 96-well plates at a density of 2 × 10^3^ cells/well (Becton Dickinson, Franklin Lakes, NJ, USA) and were concurrently treated with 0.5, 1, or 2 mM of aspirin in media containing 20, 50, or 75% unstimulated or LPS-stimulated RAW-CM and 1% FBS/DMEM for 24, 48, and 72 h. After treatment, the cells were incubated in a 0.5 mg/mL 3-(4,5-dimethylthiazol-2-yl)-2,5-diphenyltetrazolium bromide MTT (Sigma) solution for 3 h. Supernatants were aspirated, DMSO was added to solubilize the formazan crystals, and absorbance was measured at 540 nm using a spectrophotometric microplate reader (BioTek, Winooski, VT, USA). The control was considered to be 100%, and cell viability of each sample is presented as percentage of control based on the formula (*A*_sample_ − *A*_blank_)/(*A*_control_ − *A*_blank_) × 100, where *A*_sample_, *A*_blank_, and *A*_control_ refer to the absorbance of the sample, blank, and control at 540 nm, respectively.

### 2.4. Cell Migration Assay

Migration of 4T1 breast cancer cells was measured using wound-healing assays. To determine the optimal concentration of RAW-CM for 4T1 cell migration, 4T1 cells were cultured in media containing 20, 50, or 75% RAW-CM and 3% FBS/DMEM for 24 h. Cells were seeded in 24-well plates and incubated until 80% confluence was reached. This monolayer of cells was gently scratched using a 20 *μ*L pipette tip, and the media was replaced with 0.5, 1, or 2 mM aspirin in fresh medium, 50% unstimulated RAW-CM, or 50% LPS-stimulated RAW-CM for 24 h. Cells were viewed and imaged through a microscope equipped with a camera (WS500, Whited, Taoyuan, Taiwan) at 100x magnification. Then, the healing in the image was measured with a microscale of image software (Whited).

### 2.5. Cytokine Production as Measured by ELISA

The 4T1 cells, 2 × 10^4^ cells/well, were seeded in a 48-well plate overnight and then treated with 2 mM aspirin in complete medium or 50% RAW-CM for 72 h. Culture supernatants were collected, and levels of cytokines, including MCP-1 (BioLegend, San Diego, CA, USA), VEGF (Peprotech, Rocky Hill, NJ, USA), PAI-1, TNF-*α*, IL-6, and TGF-*β* (R&D, Minneapolis, MN, USA), were measured by ELISA according to the manufacturer's instructions. Briefly, plates were coated overnight with capture antibodies and then washed and blocked. After washing, the culture supernatants were added to the plates and the plates were incubated for 2 h. After washing, the plates were incubated first with detection antibodies, next with horseradish peroxidase-conjugated streptavidin, and finally with substrate solution. Absorbance was measured using a microplate reader (Molecular Devices, Sunnyvale, CA, USA). Cytokine levels were calculated based on cytokine standard curves.

### 2.6. Cocultures of 4T1 Cell and RAW 264.7 Cell

To define the role of the mammary microenvironment in tumorigenesis, the experimental models consisted of 4T1 *murine* breast cancer cells cultured alone in RAW-CM or cocultured with RAW 264.7 cells. To mimic a physiological environment where macrophages infiltrate into the areas surrounding breast cancer cells, RAW 264.7 and 4T1 cells were cocultured in the same well of 6-well plates at densities of 1 × 10^5^ and 4 × 10^5^ cells/well. The cells were then maintained in 1% FBS/DMEM and treated with 2 mM aspirin for 72 h. Culture supernatants were harvested and stored at −20°C until cytokine levels were measured by ELISA.

### 2.7. RAW 264.7 Cell Characterization

Macrophages were incubated in the presence or absence of aspirin for 72 h and cultured in either control medium, the presence of LPS for the last 24 h of the incubation, or cocultured with 4T1 cells for 72 h. To assess surface marker expression, RAW 264.7 and 4T1 cells were collected after 72 h of coculturing and stained by incubating with fluorescein FITC anti-mouse CD11c and Alexa Fluor 647 anti-mouse CD206 monoclonal antibodies (Sony Biotechnology Inc.) at 4°C in the dark for 30 min. After washing, viable cells were stained with Hoechst 33342 (ChemoMetec, Allerød, Denmark) and subjected to FlexiCyte fluorescence-activated cell sorting analysis. The frequency of cells expressing each surface marker was determined by NucleoCounter NC-3000 (ChemoMetec) and analyzed using NucleoView NC-3000 software (ChemoMetec). Expression was quantified using median fluorescence intensity for the marker of interest.

### 2.8. Statistical Analysis

Results are presented as mean ± SEM and are a compilation of at least three independent experiments. Statistically significant differences among groups were identified by one-way ANOVA with least significant difference post hoc tests using IBM Statistical Product and Service Solutions (SPSS version 19). A *p* value of less than 0.05 was considered statistically significant.

## 3. Results

### 3.1. RAW 264.7 Cell-Conditioned Media Affects 4T1 Breast Cancer Cell Viability and Migration

**Figure 1:**
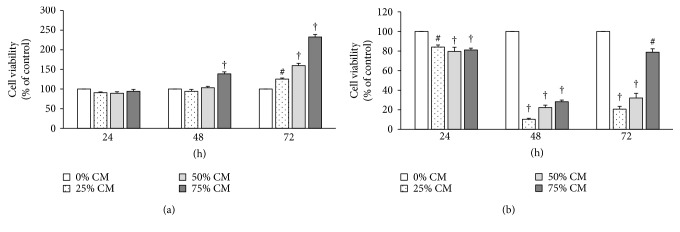
Viability of 4T1 cells cultured in different amounts of RAW 264.7 macrophage-conditioned medium (RAW-CM). Different concentrations of (a) unstimulated and (b) LPS 100 ng/mL-stimulated macrophage-conditioned medium (RAW-CM) at 25, 50, and 75% were used to culture 4T1 cells. Cells were cultured for 24, 48, and 72 h, and cell viability was measured using MTT assays. Data are from at least three independent experiments and presented as mean ± standard error of the mean (SEM). Statistical analysis was performed using one-way ANOVA and least significant difference (LSD) post hoc tests. ^#^ *p* < 0.01 and ^†^ *p* < 0.001 versus control (0% RAW-CM).

To mimic the physiological tumor environment of macrophage infiltrates into tumor tissues and to study the effect of macrophage mediators on 4T1 cell viability, breast cancer 4T1 cells were cultured in RAW 264.7 cell-conditioned media (RAW-CM), as shown in Figure 1. The 4T1 cells were cultured in different concentrations of RAW-CM in the presence or absence of lipopolysaccharide (LPS) stimulation, and cell viability was assessed using MTT assays. The culture condition lacking LPS stimulation mimicked macrophage infiltration into the breast cancer microenvironment, while the culture condition with LPS stimulation mimicked infiltrating macrophages that are active due to inflammatory responses.

A progressive increase in the number of 4T1 cells occurred with an increase in concentration of unstimulated RAW-CM. This increase in cell number, compared to the control (0% RAW-CM), occurred in a dose-dependent manner with the incubation time (Figure 1(a)), suggesting the macrophages present promoted breast cancer cell growth. The opposite result was observed when 4T1 cells were cultured in the LPS-stimulated RAW-CM, where 4T1 cell viability significantly decreased during incubations of 24 to 72 h (*p* < 0.05, Figure 1(b)). This suggests that mediators were secreted by active macrophages that caused toxicity, and thereby decreased cancer cell numbers.

**Figure 2:**
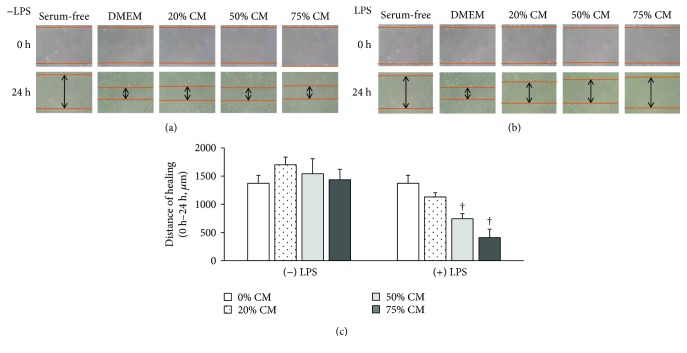
Migration of 4T1 cells cultured in different amounts of RAW-CM. Migration patterns of 4T1 cells were assessed in scratched areas by culturing cells for 24 h in 20, 50, and 75% (a) unstimulated or (b) LPS-stimulated RAW-CM and then monitoring wound healing. (c) Distance was measured by microscope under a microscale and presented as percentage inhibition relative to the control. Data are shown as mean ± SEM and are from three independent experiments. Statistical analysis was performed using one-way ANOVA and LSD post hoc tests. ^†^ *p* < 0.001 versus control (0% CM).

Wound-healing assays were used to analyze cell migration, which is an indicator of cancer metastasis. Cells were grown until a confluent monolayer and scraped, and then the distance of healing by the cell layer was measured. The 4T1 cells cultured in 3% FBS/DMEM, that is, control, exhibited apparent healing, while the cells cultured in serum-free media, that is, negative control, did not. The distance of 4T1 cell migration over 24 h was measured for each treatment condition, including cells incubated in 20, 50, and 75% RAW-CM. RAW-CM was collected from cells that were not stimulated with LPS as a spontaneous condition and was found to have no effect on cell migration (Figure 2(a)). Meanwhile, RAW-CM collected from LPS-stimulated cells inhibited healing after scraping in a dose-dependent manner (Figure 2(b)). The migration distance was measured by microscope under a microscale, and the results are shown in Figure 2(c). The 50 and 75% LPS-stimulated RAW-CM conditions significantly inhibited cell migration (*p* < 0.01), which is consistent with the effect this conditioned media had on 4T1 cell viability.

### 3.2. Aspirin Inhibited 4T1 Breast Cancer Cell Growth and Migration in RAW 264.7 Cell-Conditioned Media

**Figure 3:**
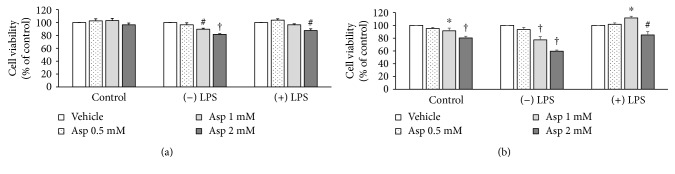
The effect of aspirin on viability of 4T1 cells cultured in RAW-CM. Different doses of aspirin were used to treat 4T1 cells cultured in 50% unstimulated or LPS-stimulated RAW-CM. Cells were cultured for (a) 24 or (b) 72 h, and cell viability was assessed using MTT assays. Data are shown as mean ± SEM and are from three independent experiments. Statistical analysis was performed using one-way ANOVA and LSD post hoc tests. ^∗^ *p* < 0.05, ^#^ *p* < 0.01, and ^†^ *p* < 0.001 versus vehicle control.

Subsequently, we investigated whether aspirin treatment influences 4T1 breast cancer cell growth when cultured under different macrophage-related conditions. The 4T1 cells were cultured in RAW-CM to mimic a microenvironment with macrophage infiltration into areas surrounding breast cancer cells, and then cell viability and migration were assessed. The 4T1 cells treated with 1 and 2 mM of aspirin had decreased cell viability when incubated in both unstimulated and LPS-stimulated RAW-CM for 24 h, while 4T1 cell numbers were not affected by aspirin in the complete medium (Figure 3(a)). Cell number displayed more apparent decreases of 23% (*p* < 0.001) and 40% (*p* < 0.001) in unstimulated RAW-CM compared to cells in control medium, when cells were treated for 72 h with 1 or 2 mM aspirin, respectively (Figure 3(b)). However, only the high dose of 2 mM aspirin inhibited cell viability in the LPS-stimulated RAW-CM.

**Figure 4:**
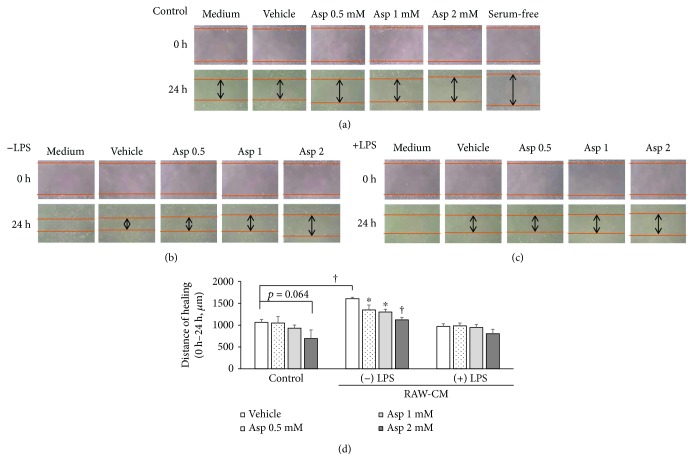
The effect of aspirin on migration of 4T1 cells cultured in RAW-CM. Different doses of aspirin were used to treat 4T1 cells, which were cultured for 24 h in (a) 3% FBS/DMEM, (b) 50% unstimulated RAW-CM, and (c) 50% LPS-stimulated RAW-CM for 24 h, and wound-healing assays were performed. (d) Distance was measured by microscope under a microscale and is presented as percentage inhibition relative to the control. Data are shown as mean ± SEM and are from three independent experiments. Statistical analysis was performed using one-way ANOVA and LSD post hoc tests. ^∗^ *p* < 0.05 and ^†^ *p* < 0.001 versus vehicle control.

To investigate the effects of aspirin on 4T1 cell migration in RAW-CM, wound-healing assays were utilized. The 4T1 cells were cultured in fresh medium (Figure 4(a)), unstimulated RAW-CM (Figure 4(b)), or LPS-stimulated RAW-CM (Figure 4(c)) to mimic the macrophage-infiltrated microenvironment. Aspirin had no effect on cell migration in the fresh medium (Figure 4(a)). In the unstimulated RAW-CM, 0.5 to 2 mM aspirin significantly delayed scratch-healing form in a dose-dependent manner (*p* < 0.05) compared to the vehicle group (Figures 4(b) and 4(d)), while healing was not affected by aspirin in the LPS-stimulated RAW-CM (Figures 4(c) and 4(d)).

Therefore, the unstimulated RAW-CM, which mimicked the tumor microenvironment, promoted growth of 4T1 cells and was suitable to use for future experiments. Meanwhile, LPS stimulation triggered RAW 264.7 cells to exert an acute inflammatory response that inhibited growth and migration of 4T1 cells. On the basis of these studies, aspirin is an effective chemopreventive agent in the tumor microenvironment but did not exert an anticancer effect during the acute inflammatory stage.

### 3.3. Aspirin Inhibited 4T1 Cell Production of Angiogenic and Inflammatory Cytokines

**Figure 5:**
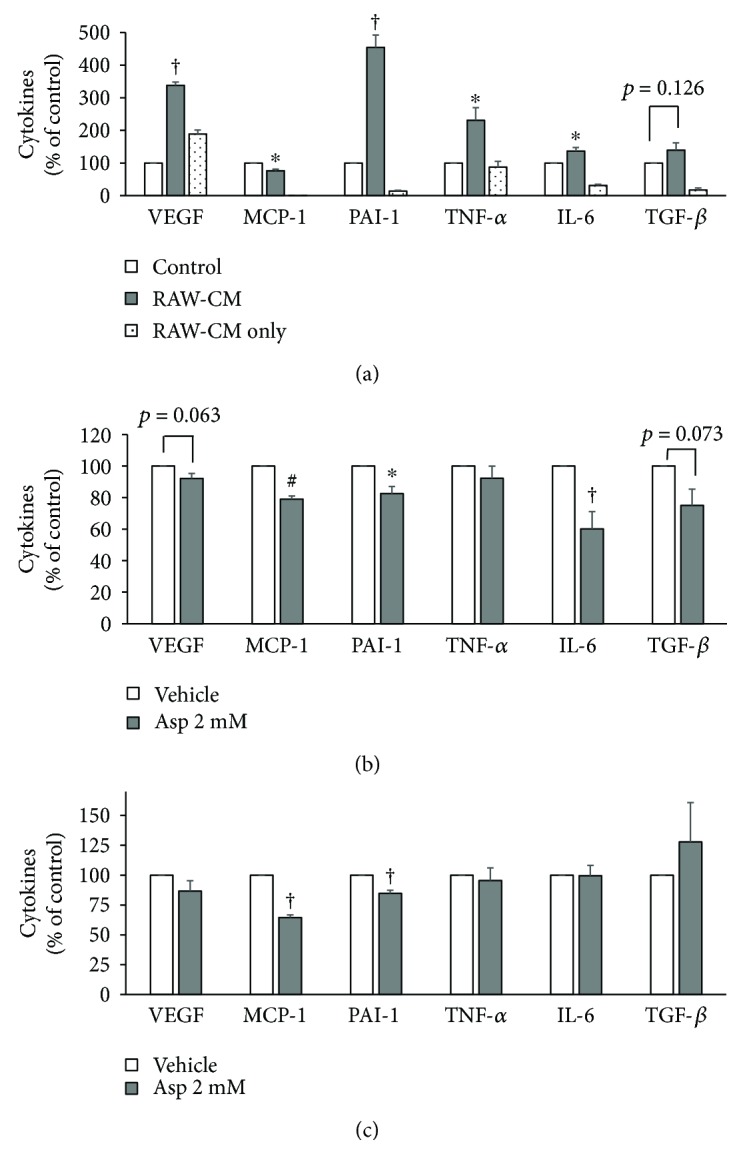
Effect of aspirin on carcinogenic cytokine production by 4T1 breast cancer cells cultured in control medium and RAW-CM. (a) Effect of RAW-CM on cytokine production of 4T1 cells. Cells were cultured in 50% RAW-CM for 72 h, and then cytokines in the supernatants were measured by ELISA. (b) Aspirin was used to treat 4T1 cells, which were cultured in control medium (1% FBS/DMEM) for 72 h, and cytokine levels in the supernatants were measured. (c) Aspirin was used to treat 4T1 cells, which were cultured in 50% RAW-CM for 72 h, and then cytokine levels in the supernatants were measured. Data are shown as mean ± SEM. Statistical analysis was performed using independent sample *t*-tests, where statistically significant differences are indicated as ^∗^ *p* < 0.05, ^#^ *p* < 0.01, and ^†^ *p* < 0.001 versus control.

Cytokines related to breast cancer carcinogenesis in the cultured supernatants were measured by ELISA. Cytokine levels are listed in Supplementary 1, and data are presented relative to the vehicle control in Figure 5. First, 4T1 cells were cultured in fresh medium (control) or RAW-CM and the supernatants were analyzed (Figure 5(a)). The RAW-CM only allowed background levels of mediators in the original conditioned medium to be measured. VEGF, plasminogen activator inhibitor-1 (PAI-1), tumor necrosis factor (TNF-*α*), and interleukin (IL-6) secretion were significantly higher when the 4T1 cells were cultured in 50% RAW-CM, suggesting that macrophage-related mediators in the conditioned media promoted carcinogenic and inflammatory cytokine production by the breast cancer cells (*p* < 0.05).

To investigate the effects of aspirin treatment on secretion of these cytokines, cytokine levels relative to tumor characteristics were analyzed (Figures 5(b) and 5(c)). As shown in Figure 5(b), when the 4T1 cells were cultured in fresh medium as a control condition, aspirin treatment significantly decreased MCP-1 (*p* = 0.001), PAI-1 (*p* = 0.019), and IL-6 (*p* < 0.001) levels and slightly decreased VEGF level (*p* = 0.063). As shown in Figure 5(c), when the 4T1 cells were cultured in 50% RAW-CM, aspirin treatment only decreased MCP-1 and PAI-1 production (*p* < 0.001 and *p* = 0.004, resp.).

### 3.4. Aspirin Regulated Macrophage Subtypes in Cocultures of Breast Cancer Cell and Macrophage

**Figure 6:**
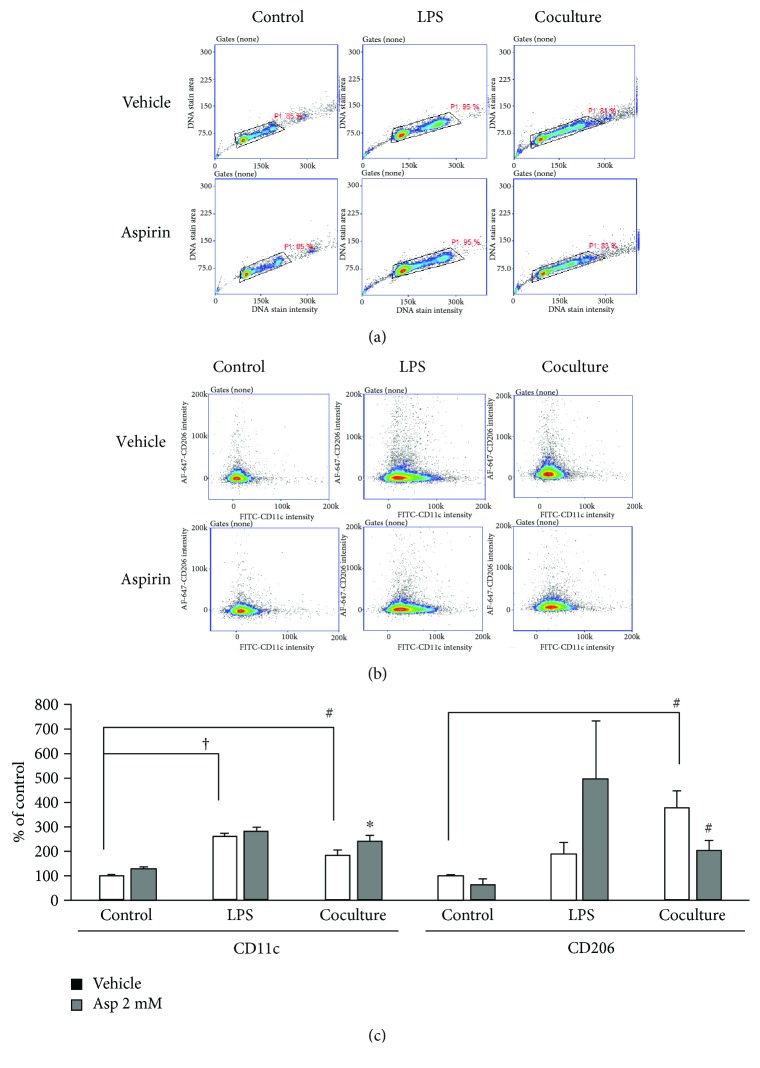
Effect of aspirin on M1 and M2 macrophage subtypes following LPS stimulation and coculture with 4T1 cells. Macrophages were incubated in the presence or absence of aspirin for 72 h and cultured in either fresh medium as a control, the presence of LPS for the last 24 h of the incubation, or cocultured with 4T1 cells for 72 h. (a) Histogram plots, (b) fluorescent intensity plots, and (c) quantitative data were presented. The immunofluorescent intensity of CD11c (M1) and CD206 (M2) on macrophages was analyzed using a NC-3000. Data are shown as mean ± SEM. Statistical analysis was performed using one-way ANOVA and LSD post hoc tests. The comparisons between different culture mediums were done by *t*-tests. Statistically significant differences are indicated as ^∗^ *p* < 0.05, #*p* < 0.01, and †*p* < 0.001 versus vehicle control.

We determined whether aspirin treatment affects M1 and M2 macrophage subpopulations based on surface marker expression. Cluster of differentiation (CD)11c is a marker of M1 macrophages, while CD206 is a marker of M2 macrophages. RAW264.7 cells were cultured in control medium, LPS-stimulated RAW-CM, or cocultured with 4T1 cells and then characterized. Histograms and fluorescence intensity plots are presented in Figures 6(a) and 6(b), while quantitative data is presented in Figure 6(c). CD11c expression increased by 181% in RAW 264.7 cells following LPS stimulation (*p* < 0.001), but CD206 marker expression was not affected. When RAW 264.7 cells were cocultured with 4T1 breast cancer cells, CD206 expression significantly increased by 281% (*p* = 0.002). After treatment with aspirin, CD11c significantly increased by 32% (*p* = 0.012) and CD206 decreased by 41% (*p* = 0.046) compared to the vehicle control in cocultured RAW 264.7 cells, suggesting aspirin altered the macrophage prolife when in the presence of neoplastic cells, but not the condition of LPS stimulation (Figure 6(c)).

### 3.5. Aspirin Inhibits Crosstalk and Production of Carcinogenesis-Related Cytokines in Cocultures of Breast Cancer Cell and Macrophage

**Figure 7:**
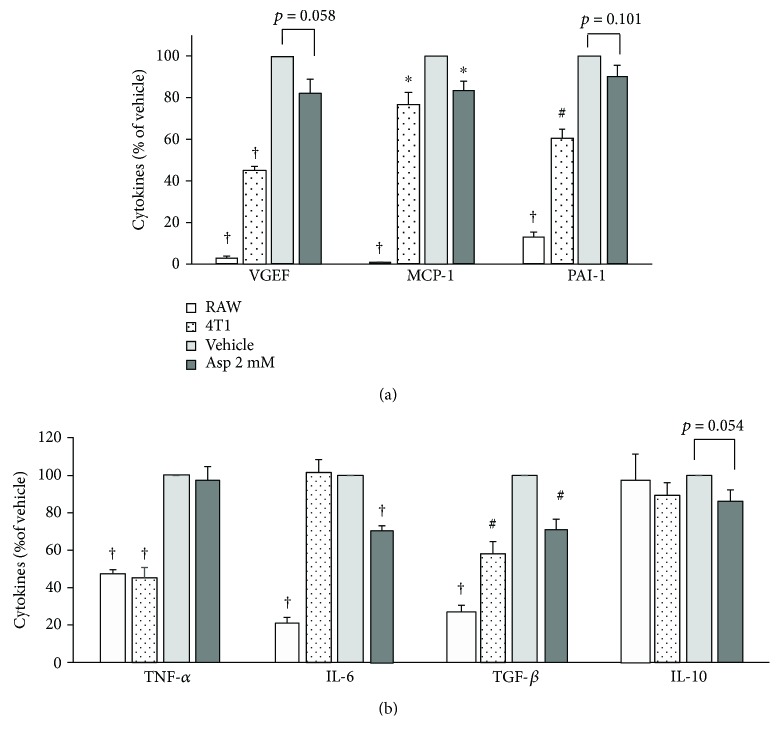
Aspirin inhibited angiogenic and inflammatory cytokines in supernatants of 4T1 and RAW 264.7 cell cocultures. 4T1 cells were cultured in the presence of macrophages for 72 h, supernatants were collected, and cytokine levels relative to tumor characteristics were measured by ELISA. (a) Angiogenic cytokines VEGF, MCP-1, and PAI-1. (b) Inflammation-related cytokines TNF-*α*, IL-6, TGF-*β*, and IL-10. The blank bar indicates RAW 264.7 cells only, the dotted bar indicates 4T1 cells only, the gray bar indicates cocultures containing both cells, and dark gray indicates cocultures treated with 2 mM aspirin. Data are shown as mean ± SEM. Statistical analysis was performed using independent sample *t*-tests. Statistically significant differences are indicated as ^∗^ *p* < 0.05, ^#^ *p* < 0.01, and ^†^ *p* < 0.001 for treatment versus co-control vehicle.

To further confirm the production of potential mediators of interactions between cells in culture supernatants, 4T1 and RAW 264.7 cells were cocultured together to mimic the physiology of the tumor microenvironment. Cytokine levels are listed in Supplementary 2, and the data are presented relative to the vehicle control in Figure 7. Cytokine levels relative to tumor characteristics were assessed for VEGF, MCP-1, PAI-1, TNF-*α*, IL-6, transforming growth factor- (TGF-) *β*, and IL-10 by ELISA at the end of 72 h of coculture. There were only very low levels of VEGF, MCP-1, PAI-1, TNF-*α*, and TGF-*β* in the individual RAW 264.7 or 4T1 cell supernatants. When both cell types were present and treated with 2 mM aspirin, there was significant inhibition of MCP-1, IL-6, and TGF-*β* (*p* = 0.019, *p* < 0.001, and *p* = 0.008, resp.), and trending decreases in VEGF, PAI-1, TNF-*α*, and IL-10 (*p* = 0.058, *p* = 0.101, *p* = 0.058, and *p* = 0.054, resp.).

**Figure 8:**
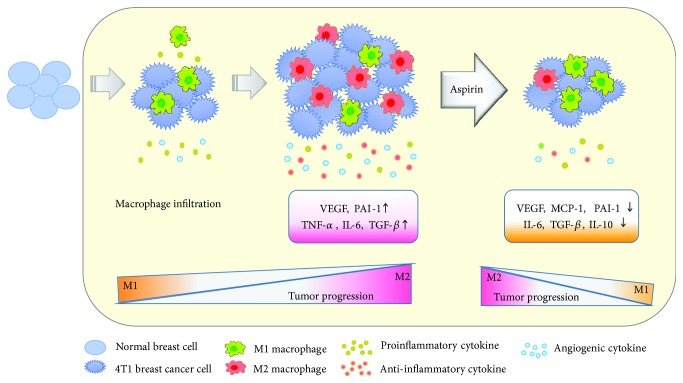
The schema of possible mechanism of chemoprevention of aspirin. In 4T1 breast cancer cell environment, RAW264.7 macrophage infiltration increased VEGF, PAI-1, TNF-*α*, IL-6, and TGF-*β* levels, and M2 macrophage expression, resulting to, benefit to tumor progression. Aspirin treatment decreased angiogenic and inflammation-associated cytokine VEGF, PAI-1, MCP-1, IL-6, IL-10, and TGF-*β* production. In addition, treatment of aspirin increased M1 expression and decreased M2 expression in macrophages, resulting to interference of the communication in this microenvironment and blunted tumor progression.

The effects of aspirin treatment in the coculture model were apparent compared to the RAW-CM model, suggesting cocultures containing both types of cells can effectively crosstalk. These data indicate that aspirin disrupted secretion of mediators associated with carcinogenesis in both RAW-CM and cocultures. A schematic of factors with a possible active role in aspirin treatment is proposed in Figure 8.

## 4. Discussion

Breast cancer is the most prevalent malignant tumor currently found in women. The breast tumor microenvironment includes neoplastic, neighboring stromal, and recruited immune cells, such as macrophages and lymphocytes, where crosstalk among these cells is involved in tumor progression and metastasis [2]. Interestingly, macrophages, the most abundant immune cell type present in solid tumors, infiltrate and secrete many cytokines while neoplastic cells form. This creates chronic inflammation that provides conditions in this microenvironment conducive to tumor development and angiogenesis [17, 18].

The breast cancer cell line 4T1 is triple-negative, which is a form of breast cancer associated with a poor prognosis because the cells lack effective therapeutic targets, behave aggressively, and are accompanied with overexpression of inflammation-related mediators [15]. This has motivated scientists to identify effective agents against this type of cancer. In this present study, aspirin was determined to be a potential chemopreventive agent with antiangiogenic and anti-inflammatory properties in a tumor microenvironment created using RAW-CM and cocultures of RAW 264.7 macrophages and 4T1 breast cancer cells. The results of the present study suggest aspirin interfered with crosstalk between these two cell types and, thus, inhibited cancer cell growth and migration.

Normally, macrophages have a critical role in host defense that involves connecting innate and adaptive immune responses, as well as tissue repair. Macrophages secrete multiple cytokines that participate in inflammatory responses, tissue damage, pathogen clearance, tissue homeostasis, and disease development [19, 20]. LPS, that is, bacterial endotoxin, is a common agent that activates macrophages involved in the innate immune response and causes immune cell infiltration and inflammation [21, 22]. A number of studies have shown that endotoxin may be anticarcinogenic, possibly due to its ability to recruit and activate immune cells and proinflammatory mediator production [22]. Tumorigenesis accompanies macrophage infiltration. Therefore, RAW-CM may mimic the microenvironment associated with chronic disease, including the presence of multiple inflammatory mediators [17]. In the RAW-CM model, LPS stimulation triggered RAW 264.7 cells to undergo an acute inflammatory response and, thus, inhibit 4T1 cell growth and migration, which is consistent with other evidence. LPS activates TLR4 signaling in tumor cells, leading to tumor evasion from immune surveillance and tumor growth delay [23]. Meanwhile, unstimulated RAW-CM, which may mimic the tumor microenvironment, promoted 4T1 cell growth. This suggests that aspirin is a promising chemopreventive agent and it is not only anti-inflammatory but also anticarcinogenic. These anticancer properties have also been exhibited in human breast cancer MDA-MB-231 cells [24].

In a previously published study, mice were inoculated with 4T1 cells and implanted with sponge discs for 1 or 24 days to create acute and chronic inflammatory environments [25]. Tumor progression and circulating levels of VEGF and TNF-*α* were greater in the presence of chronic inflammation than acute inflammation. In addition, VEGF and TNF-*α* molecules are critical for the proliferation, angiogenesis, macrophage recruitment, and metastasis associated with tumor progression [25]. Populations of macrophages, dendritic cells, and lymphocytes were significantly larger in mice with chronic inflammation [25], suggesting that chronic cell infiltration is important for tumor progression. In an obesity-related breast cancer study, 4Tl cell proliferation was significantly observed when cells were cultured in adipocyte-conditioned medium without any stimulation, indicating that spontaneous adipocyte infiltration contributed to 4T1 cell growth [16].

Our previous study demonstrated that aspirin treatment significantly inhibits the proliferation and migration of 4T1 cells, as well as causes an associated decrease in MCP-1 and VEGF production [26]. In this present study, PAI-1 and IL-6 production by 4T1 cells was also inhibited by aspirin treatment. In the RAW-CM model, VEGF, PAI-1, TNF-*α*, and IL-6 production by 4T1 cells significantly increased, indicating there are carcinogenic mediators in the RAW-CM. After aspirin treatment, production of MCP-1 and PAI-1 decreased, suggesting that aspirin interfered with interactions between macrophages and breast cancer cells and, thus, inhibited tumorigenic signals. Moreover, in an obesity-related breast cancer study involving 4T1 cells cultured in 3T3-L1 adipocyte-conditioned medium and cocultured with adipocytes, aspirin decreased the production of MCP-1 and PAI-1 [26]. This is consistent with the data from this present study, supporting that these two cytokines have important roles in immune cell recruitment and tumor progression.

MCP-1, that is, CCL-2, is a chemokine that recruits and activates monocytes during inflammation. In tumor progression, MCP-1 plays an important role through facilitation of macrophage infiltration, which is involved in tumor progression and immunosurveillance [27, 28]. In addition, a previous study reported that blocking MCP-1 signaling notably inhibited 4T1 cell migration [29]. PAI-1 is produced by multiple cells and is involved in several pathological conditions, including aging, obesity, and inflammation, and high levels have been demonstrated to accompany tumor progression [30]. Recently, TGF-*β*-treated endothelial cells were reported to induce PAI-1 secretion and promote metastasis of triple-negative breast cancer cells [31], illustrating the potential of PAI-1 as a target of breast cancer therapies. In addition, IL-6 and TNF-*α* are conductor cytokines that mediate and have multiple physiological functions in various pathogenic inflammatory diseases, where they are involved in tumor progression, angiogenesis, and migration [32]. Recently, it was revealed that proinflammatory cytokines in serum, such as IL-6, IL-8, and TNF-*α*, are associated with clinical stage and lymph node metastasis in breast cancer patients [32]. The levels of these cytokines are associated with the course of breast tumorigenesis, and, thus, these cytokines have potential as prognostic cancer biomarkers.

In this present study, aspirin suppressed MCP-1, PAI-1, and IL-6 production by 4T1 cells cultured in fresh medium and RAW-CM, suggesting to inhibit proliferation and migration of breast cancer cells. In the coculture model, treatment with aspirin significantly inhibited MCP-1, IL-6, and TGF-*β* and slightly inhibited VEGF, PAI-1, TNF-*α*, and IL-10 production. Production of these inflammatory and angiogenic mediators by 4T1 cells in fresh medium, RAW-CM, and coculture models was blocked by aspirin. On the basis of these results, the suppressive properties of aspirin interfere with community-associated factors in the breast tumor microenvironment. In addition, aspirin may also act through other pathways to exert its chemopreventive properties involving inflammation, cyclooxygenase- (COX-) 2, platelets, hormones, or PI3 kinase [33]. One of the most studied aspirin anticancer mechanisms is the partially downregulated COX-2 expression in many types of breast cancer cells, including MCF-7, MDA-MB-231, and SK-BR-3, contributing to inhibition of cancer cell proliferation [34].

Macrophages can divide into two distinct phenotypes of M1 and M2. M1 macrophages are promoted by T-helper cell type 1 (Th1) cytokines and produce proinflammatory cytokines that evoke an adaptive immune response. Meanwhile, Th2 cytokines polarize monocytes into M2 macrophages that promote angiogenesis, clean injured tissues, and suppress adaptive immune responses [7]. Imbalances in M1 and M2 macrophage populations may lead to pathological changes [35]. It has been demonstrated that mice that received 7,12-dimethylbenz(a)anthracene chemical carcinogens have higher F4/80+ macrophage recruitment in perigonadal adipose tissue compared to mice that did not receive any carcinogen, especially, the higher level of CD11c + M1 type [36]. In the present study, there was a significant increase in M2 cells when RAW 264.7 cells were cocultured with 4T1 cells, suggesting that this suppressive microenvironment promoted the growth of breast cancer cells. In the tumor microenvironment, malignancies recruit circulating monocytes that have differentiated into TAMs. TAMs resemble M2 macrophages and exert protumor functions through immunosuppressive actions [5]. Therefore, modifications, such as through suppression of TAM recruitment, switching of the TAM phenotype, and production of associated mediators, have been proposed as cancer therapeutic strategies [37].

Interestingly, aspirin treatment increased M1 marker expression, but decreased M2 marker expression in cocultures of the present study, suggesting that aspirin influences the macrophage profile in the neoplastic microenvironment away from a suppressive immune response, thus contributing to breast cancer cell suppression. Recently, it was demonstrated that macrophage phenotypes are regulated by aspirin in a model of RAW 264.7 cells cultured in pancreatic cancer cell line Panc02-conditioned medium. Aspirin significantly decreased protein and RNA levels of the M2 marker CD206 and prevented pancreatic carcinogenesis [38]. Burnett and colleagues reported that aspirin upregulates IL-10 gene expression in THP-1 cells, but not in cocultures of MCF-7 and THP-1 cells [39]. In a clinical trial on breast cancer patients, TGF-*β* expression was lower during the early stages of disease, but higher and associated with CCL2 levels during late stages. Moreover, TGF-*β* stimulated CCL2 expression and then induced monocytes/macrophages to secrete Th2-attracting chemokines into a breast cancer MDA-MB-231 cell tumor microenvironment [40]. In the present study, aspirin inhibited TGF-*β* expression in the coculture model, resulting in decreases in MCP-1 production and Th2 accumulation that dampened downstream communication in the microenvironment.

Clinical trials have revealed that aspirin is an effective chemopreventive agent. Observational studies have shown that regular aspirin use reduces the incidences of several cancers, as well as distant metastases of these cancers [41]. Meta-analyses and systematic reviews have also proposed that aspirin's chemopreventive properties can be used to fight breast cancer [13, 14]. In cardiovascular subjects of five large randomized trials, aspirin use decreased the risk of cancer mortality and metastases [33]. Recently, a larger cohort study that included 13 prospective studies with 857,831 subjects revealed that long-term (>5 years) regular use of aspirin 2 to 7 times/week prevented breast cancer [42]. Based on previous findings, regular use of aspirin (75 to 350 mg/day) reduces the incidence of and mortality from breast cancer in epidemiologic experiments [13, 14, 33, 42]. Researchers need to pursue a comprehensive understanding of aspirin treatment-associated issues, such as gastrointestinal side effects, optimal doses, duration, and combinations with other compounds, to facilitate the use of aspirin as a cancer therapy.

## 5. Conclusions

Based on accumulating evidence, macrophages play a crucial role in the tumor microenvironment, which includes intricate crosstalk involving a series of inflammatory chemokines and cytokines and angiogenic mediators secreted from neoplastic cells and infiltrating macrophages. The findings of this study indicate that aspirin has chemopreventive properties that function through both 4T1 breast cancer cells and macrophages. Aspirin interfered with the connection between various cells by decreasing communication through proinflammation and angiogenic mediators and modulating M1/M2 macrophage subtypes, suggesting that aspirin is a promising agent to prevent tumor progression.

## Data Availability

The data used to support the findings of this study are all provided in the manuscript and supplementary file.

## Supplementary Materials

Supplementary Materials Supplementary 1: effect of aspirin on carcinogenic cytokine production by 4T1 breast cancer cells cultured in control medium and RAW-CM. Supplementary 2: aspirin inhibited angiogenic and inflammatory cytokines in supernatants of 4T1 and RAW 264.7 cell cocultures.
