# Supplementary material for: Aspirin Disrupts the Crosstalk of Angiogenic and Inflammatory Cytokines between 4T1 Breast Cancer Cells and Macrophages
Source: Mediators Inflamm. 2018 Jun 24;2018:6380643. doi: 10.1155/2018/6380643 (PMC6035832; doi:10.1155/2018/6380643)
Supplement: Supplementary Materials — Supplementary 1: effect of aspirin on carcinogenic cytokine production by 4T1 breast cancer cells cultured in control medium and RAW-CM. Supplementary 2: aspirin inhibited angiogenic and inflammatory cytokines in supernatants of 4T1 and RAW 264.7 cell cocultures. [file 6380643.f1.docx]

Supplementary 1. Effect of aspirin on carcinogenic cytokine production by 4T1 breast cancer cells cultured in control medium and RAW-CM ^a^

|  | Angiogenetic cytokines | | |
| --- | --- | --- | --- |
|  | VEGF (ng/mL) | MCP-1 (ng/mL) | PAI-1 (ng/mL) |
| Control medium^b^ |  |  |  |
| Vehicle | 1.7 ± 0.1 | 10.4 ± 1.0 | 1.0 ± 0.1 |
| Aspirin | 1.6 ± 0.1 | 8.8 ± 0.9 | 0.8 ± 0.1 |
| RAW-CM^b^ |  |  |  |
| Vehicle | 5.8 ± 0.3* | 7.8 ± 0.6 | 4.7 ± 0.8* |
| Aspirin | 4.9 ± 0.1^#^ | 5.3 ± 0.5^#^ | 4.0 ± 0.7 |
| RAW-CM only | 3.2 ± 0.1 | 0.13 ± 0.01 | 0.13 ± 0.01 |
|  | Inflammatory cytokines | | |
|  | TNF-α (pg/mL) | IL-6 (pg/mL) | TGF-β (pg/mL) |
| Control medium |  |  |  |
| Vehicle | 128.3 ± 18.3 | 74.2 ± 8.8 | 75.2 ± 10.0 |
| Aspirin | 113.9 ± 11.6 | 43.7 ± 3.5^#^ | 58.8 ± 12.4 |
| RAW-CM |  |  |  |
| Vehicle | 275.7 ± 27.8* | 101.0 ± 13.8 | 102.8 ± 21.4 |
| Aspirin | 251.6 ± 13.4 | 96.6 ± 8.2 | 112.1 ± 15.5 |
| RAW-CM only | 101.5 ± 7.1 | 21.7 ± 0.4 | 15.5 ± 5.8 |

^1^ Data was showed as mean ± SEM, and statistical analysis was assayed by independent sample t-test, significantly difference at *p<0.05 as vehicle group in RAW-CM vs. vehicle group in Control medium; #p<0.05 as aspirin vs. vehicle group.

^b^ 4T1 cells were cultured in control medium or 50% RAW-CM and treated with 2 mM aspirin for 72 h.

Supplementary 2. Aspirin inhibited angiogenic and inflammatory cytokines in supernatants of 4T1 and RAW 264.7 cell co-cultures ^a^

|  | | Angiogenic cytokines | | | | | | | | | |
| --- | --- | --- | --- | --- | --- | --- | --- | --- | --- | --- | --- |
|  | | VEGF (ng/mL) | |  |  | MCP-1 (ng/mL) | |  |  | PAI-1 (ng/mL) | |
| RAW only | | 0.18 ± 0.0* | |  |  | 0.16 ± 0.0* | |  |  | 0.25 ± 0.0* | |
| 4T1 only | | 2.7 ± 0.4* | |  |  | 12.3 ± 0.6* | |  |  | 1.2 ± 0.1* | |
| Co-culture | |  | |  |  |  | |  |  |  | |
| Vehicle | | 6.2 ± 0.5 | |  |  | 16.1 ± 0.7 | |  |  | 2.0 ± 0.2 | |
| Aspirin^b^ | | 5.1 ± 0.5 | |  |  | 13.4 ± 0.6* | |  |  | 1.8 ± 0.2 | |
|  | Inflammatory cytokines | | | | | | | | | | |
|  | TNF-α (pg/mL) | | IL-6 (pg/mL) | | | | TGF-β (pg/mL) | | | | IL-10 (pg/mL) |
| RAW cell only | 202.1 ± 7.3* | | 62.2 ± 0.9* | | | | 106.6 ± 16.2* | | | | 223.0 ± 13.7 |
| 4T1 cell only | 194.3 ± 25.8* | | 315.1 ± 29.6 | | | | 233.6 ± 39.4* | | | | 208.7 ± 15.3 |
| Co-culture |  | |  | | | |  | | | |  |
| Vehicle | 428.0 ± 15.4 | | 320.8 ± 43.7 | | | | 396.1 ± 31.2 | | | | 236.2 ± 14.5 |
| Aspirin | 413.6 ± 29.5 | | 226.8 ± 33.7 | | | | 279.4 ± 31.3* | | | | 206.1 ± 22.7 |

^a^ Data was showed as mean ± SEM, and statistical analysis was assayed by independent sample t-test, significantly difference at * p < 0.05 as treatment vs. co-control vehicle group.

^b^ 4T1 cells were cultured with macrophages and treated with 2 mM aspirin for 72 h.
